# Tuberculosis Co-Infection Is Common in Patients Requiring Hospitalization for COVID-19 in Belarus: Mixed-Methods Study

**DOI:** 10.3390/ijerph19074370

**Published:** 2022-04-05

**Authors:** Yuliia Sereda, Oleksandr Korotych, Dzmitry Klimuk, Dzmitry Zhurkin, Varvara Solodovnikova, Malgorzata Grzemska, Viatcheslav Grankov, Hennadz Hurevich, Askar Yedilbayev, Alena Skrahina

**Affiliations:** 1 World Health Organization Regional Office for Europe, Marmorvej 51, DK-2100 Copenhagen, Denmark; yulia.v.sereda@gmail.com (Y.S.); gosiagrzemska@gmail.com (M.G.); yedilbayeva@who.int (A.Y.); 2 Republican Scientific and Practical Centre for Pulmonology and Tuberculosis, 157 Dolginovskij trakt, 220053 Minsk, Belarus; dzklm99@yahoo.com (D.K.); dmitry_zhurkin@yahoo.com (D.Z.); varvaras@tut.by (V.S.); ge.gurev@gmail.com (H.H.); alena.skrahina@gmail.com (A.S.); 3 World Health Organization Country Office in Belarus, Fabriciusa str. 28 (Room 401), 220007 Minsk, Belarus; grankovv@who.int

**Keywords:** tuberculosis, rifampicin resistance, Xpert MTB/RIF, COVID-19, case-finding, Eastern Europe, operational research

## Abstract

A significant drop in tuberculosis (TB) case-finding has been widely reported during the period of the COVID-19 pandemic. To address a decrease in TB notification, Belarus introduced laboratory TB testing in patients with the laboratory-confirmed coronavirus disease 2019 (COVID-19). We conducted a secondary analysis of health records among 844 patients with laboratory-confirmed COVID-19 diagnosis who were admitted to repurposed departments at TB hospitals and who were tested by Xpert MTB/RIF (Cepheid Inc., Sunnyvale, CA, USA) in five Belarus regions between April and October 2021. Quantitative analysis followed by 13 individual interviews with health managers, physicians, and nurses participating in the intervention. Most patients were male (64%) and mean age was 43.5 ± 16 years. One in twenty (n = 47, 5.6%) patients were co-infected with active pulmonary TB, and over one-third of them (n = 18) had rifampicin resistance. In-hospital mortality was comparable in patients with and without TB co-infection (2.1% and 2.3% respectively, *p* > 0.99). Laboratory TB testing among patients with COVID-19 at repurposed departments of TB hospitals is feasible in Belarus and may improve TB case-finding.

## 1. Introduction

Tuberculosis (TB) was a major cause of death from a single infectious agent before the escalation of coronavirus disease 2019 (COVID-19), a novel disease caused by severe acute respiratory syndrome coronavirus 2 (SARS-CoV-2) [1]. The relationship between these two diseases is bidirectional. Immunosuppression induced by TB may increase the susceptibility to COVID-19, and vice-versa [2]. People with TB are more likely to experience severe COVID-19 progression [3]. Both diseases are subject to airborne transmission, share similar symptoms of cough, fever, and breathlessness and analogous risk factors, such as poverty, overcrowding, and diabetes [2,4]. Both TB and COVID-19 are affected by stigma and shame that may discourage people from seeking care until they are seriously ill and from collaborating in contact investigations [5].

In addition, there are indirect effects of COVID-19 pandemic on the diagnosis of TB. Reduced attendance at TB diagnostic services and reassignment of TB personnel have been widely reported [6]. Many countries observed a significant drop in presumptive and confirmed TB cases relative to the period before the COVID-19 pandemic [5]. Simulation studies in India, Kenya, and Ukraine showed that COVID-19 related lockdowns can cause long-lasting effects on TB burden, such as an increase in TB incidence by up to 9% and an increase in TB deaths by up to 16% over the next five years [7]. Furthermore, the disruptive effect of COVID-19 pandemic on TB services may slow down countries’ progress in reaching the goals of the ‘End TB Strategy’ by 2030, which aims to reduce TB incidence by 80%, to decrease TB-related deaths by 90%, and to eliminate catastrophic costs for TB-affected households [1,8].

Belarus is one of 18 priority countries in the World Health Organization (WHO) European Region to combat TB, and one of the 30 countries with the highest burdens of rifampicin-resistant and multidrug-resistant TB (MDR/RR-TB) in the world [1]. In 2020, the estimated TB incidence in the country was 2500 or 26 per 100,000 population [1]. Prevalence of MDR/RR-TB in Belarus is among the highest in the world, reaching 49% among notified TB cases. [1] About 9% of patients with TB are also co-infected with HIV [1]. The COVID-19 pandemic has had a major impact on the work of the Belarus TB program. An average 9% annual decrease in TB notification has been observed in Belarus during 2015–2019 [1]. However, a 32% decrease in TB notification has been observed in 2020 compared to 2019 [1].

The first case of COVID-19 in Belarus was registered on 24 February 2020 [9]. Contrary to other countries in the region, a national lockdown has never been implemented in Belarus. By the end of 2021, more than 685,000 cases have been confirmed since the beginning of the pandemic [9]. National regulatory bodies approved four COVID-19 vaccines for use in Belarus: Sputnik V and Sputnik Light (Russian Federation, Gamaleya), KoviVac (Russian Federation, Chumakov Center), and Covilo (China, Sinopharm) [10]. Vaccinations started in January 2021. By the end of 2021, COVID-19 vaccine uptake was 52% with at least one dose and 38% for the completed series [11].

In May 2020, the WHO released an Information Note on TB and COVID-19 urging national TB programs to maintain continuity of essential TB services during the COVID-19 pandemic. To address the drop in TB case-finding, the document recommends simultaneous testing for both TB and COVID-19 when: (a) there are clinical features that are common to both diseases; (b) simultaneous exposure to both diseases has been reported; or (c) there is a presence of a risk factor for poor outcomes to either disease [8]. Belarus is one of the first countries in Europe that introduced laboratory TB screening in patients with COVID-19. In March 2020, TB hospitals in all regions of the country allocated beds and dispatched professional staff for COVID-19 services. Since April 2021, TB hospitals began laboratory TB testing using Xpert MTB/RIF (Cepheid Inc., Sunnyvale, CA, USA) among patients with confirmed COVID-19 and chest X-ray lung abnormalities who were admitted to repurposed departments of TB hospitals. The aim of our study was to evaluate outcomes and feasibility of this intervention. The study objectives were to estimate the proportion of patients with laboratory-confirmed TB coinfection among patients with COVID-19, and to compare in-hospital mortality with and without TB co-infection. Additionally, we aimed to identify barriers affecting implementation of laboratory TB testing in patients with COVID-19. We did not have a prespecified hypotheses for the study objectives.

## 2. Materials and Methods

### 2.1. Design

The study comprised a mixed-method design, which combined a secondary analysis of patient records (quantitative study) and individual interviews among health workers (qualitative study).

### 2.2. Study Sites

Patients with COVID-19 underwent Xpert MTB/RIF (Cepheid Inc., Sunnyvale, CA, USA) testing in five out of seven regional civilian TB hospitals (i.e., hospitals in the country capital (Minsk city) and four regions (Homel’, Hrodna, Mahilëu, and Vicebsk)). We included all these sites into the study. The intervention has not been implemented in the Brest and Minsk regions or TB hospitals in penitentiary services (prisons).

All study sites were equipped with laboratories performing smear microscopy, molecular methods, culture, and drug susceptibility testing of first- and second-line anti-TB drugs. The TB hospital in Minsk city had a national TB reference laboratory (level IV), and regional TB hospitals had level III TB reference laboratories.

TB hospitals in Hrodna, Mahilëu, and Minsk city started the intervention in April 2021. However, the intervention was interrupted after 2.5 weeks at the Mahilëu hospital because the allocated GeneXpert system (Cepheid Inc., Sunnyvale, CA, USA) was not functioning properly and could not be repaired or replaced during the study period. In August 2021, the TB hospital in Homel’ region began implementing the intervention, and the hospital in Vicebsk region joined a month later.

The proportion of repurposed beds in enrolled TB hospitals ranged from 19 to 57%, with a smaller proportion in regions with high TB burden (Table 1). During the first months of COVID-19 pandemic, TB hospitals admitted patients with COVID-19 when general hospitals (polyclinics) and infectious diseases hospitals experienced a shortage of beds. Later, TB hospitals could admit patients with COVID-19, disregarding bed occupancy in other facilities.

### 2.3. Laboratory TB Testing

TB diagnostic algorithm in the country follows the WHO recommendations [12]. Xpert MTB/RIF (Cepheid Inc., Sunnyvale, CA, USA), a cartridge-based nucleic acid amplification test (NAAT), was introduced in 2012 and serves as the point of care fist diagnostic test for presumptive TB cases per national diagnostic algorithm. All study sites utilized MTB/RIF Ultra cartridges. One sputum sample was collected per person. Health workers followed standard operational procedures for sputum collection [13]. Under certain clinical circumstances, particularly among patients without productive cough, health workers practiced sputum induction. Patients with laboratory-confirmed TB were linked with TB physicians for further diagnosis and management of TB.

### 2.4. Quantitative Study

#### 2.4.1. Participants, Period, and Sample

We included patients with laboratory-confirmed COVID-19 diagnosis (U07.1 code, ICD-10, 2019 edition) who were admitted to repurposed TB hospitals and underwent Xpert MTB/RIF (Cepheid Inc., Sunnyvale, CA, USA) testing during COVID-19 treatment in Belarus between April and October 2021. We excluded patients who had active TB and received TB treatment at the time of COVID-19 diagnosis. There was no sampling in the quantitative study. We included health records of all eligible patients within the study period.

#### 2.4.2. Data Sources and Variables

Following the admission of patients with COVID-19 to repurposed departments of TB hospitals, a case-based paper form 003y-07 “Inpatient health record” was completed. Information on demographics, comorbidities, and TB history from this form was entered into the data frame designed for the study in MS Excel. Results of TB testing were extracted from the electronic laboratory database and matched with other data by patient ID. Our primary outcome was laboratory-confirmed TB disease defined as a positive result of Xpert MTB/RIF (Cepheid Inc., Sunnyvale, CA, USA). Secondary outcomes were rifampicin resistance confirmed by Xpert and in-hospital mortality. Potential predictors of TB and COVID-19 coinfection included study site, age in intervals (country programmatic definition), sex, residence (urban/rural), employment (employed/unemployed/other), body mass index (BMI), and comorbidities.

#### 2.4.3. Data Analysis

Demographic and clinical characteristics of the study participants were summarized with frequencies and proportions for categorical variables and ‘five-number’ summaries for numeric variables (mean, standard deviation, median, interquartile range, and range). Of those tested by Xpert MTB/RIF (Cepheid Inc., Sunnyvale, CA, USA), we estimated the proportion of patients with detected MTB and proportion of patients with rifampicin resistance and respective 95% confidence intervals. As a sensitivity analysis, we calculated the proportion of culture-confirmed TB disease of those tested by Xpert using Lowenstein-Jensen medium. We compared the proportion of in-hospital mortality among patients with and without MTB detected by Fisher Exact Test. Binary and adjusted linear probability models were used to test proportion differences in COVID-19 and TB co-infection by region, age groups, sex, type of residence, employment, history of TB treatment, known exposure to a person with TB before admission, and comorbidities. A predictive approach was used in our modeling. We chose linear probability models instead of logistic regression models considering the intuitive interpretation of estimates and that they do not suffer from complete/quasi-complete separation and bias due to rare events. We performed a purposeful selection model building strategy by consequently testing the effect of additional variables on the overall model fit using analysis of variance and information criteria.

Data analysis was conducted in R version 3.5.2 (The R Foundation for Statistical Computing).

### 2.5. Qualitative Study

#### 2.5.1. Theoretical Framework

We used phenomenology as a theoretical framework to focus on individual experiences of health workers related to the implementation of the intervention.

#### 2.5.2. Participants

A qualitative study was conducted in four TB hospitals (Homel’, Hrodna, Vicebsk regions, and Minsk city). We did not include Mahilëu’s TB hospital, considering that the site stopped the intervention shortly after the study’s start. We purposely selected four TB hospital managers, four TB physicians, and four TB nurses who were engaged in treatment of patients with COVID-19 and participated in laboratory TB testing (one per site for each profession). Additionally, we enrolled two representatives of the National TB Programme. In total, we conducted 13 of 14 expected individual interviews. One health manager was on sick leave during the period of data collection. The sample size of the qualitative study was designed considering a small number of study sides, limited number of health workers providing laboratory TB testing in patients with COVID-19 at these sites, time constraints, and anticipated difficulty of accessing health workers involved in COVID-19 response. We did not conduct interviews with patients with COVID-19.

#### 2.5.3. Data Collection Procedures

The principal Investigator (Alena Skrahina, female-identifying, MD) of the study, a representative of the National TB Programme in Belarus, identified health workers for the interviews. Recruitment prioritized health workers who had extensive experience in TB service provision (≥3 years) and who were eager to communicate and share their own opinion. Permission of the health workers supervisor was obtained prior to contacting them for the interview. Alena Skrahina approached identified health workers by phone call or email. The invitation stressed the voluntary nature of participation in the study. Potential participants were referred to Yuliia Sereda (female-identifying, PhD in Sociology), a WHO Regional Office for Europe consultant. She facilitated informed consent process and interviews. Yuliia Sereda did not have any relationship with the participants of the qualitative study. Participants did not have any prior knowledge about the interviewer, except for the occupation at the time of the study. Interviews were conducted remotely via Zoom, and there were no other people besides the participant and the interviewer. Participants could choose the time and date of the interview within the qualitative study period (December 2021). There were not rescheduled interviews. No compensation was offered for the participants of the qualitative study.

A list of questions used for the individual interviews was developed based on a literature review and discussions with the representatives of the National TB Programme in Belarus (Supplementary Materials, Table S1). The guide was pilot-tested by the National team (Dzmitry Zhurkin, MD and Varvara Solodovnikova, MD). Information obtained via the individual interviews was audio-recorded for the purpose of transcribing the interview to a MS Word file, after which the audio was deleted. Yuliia Sereda made fields notes during the interviews to describe issues if any (i.e., poor connection, background noise, low conversation dynamic, etc.) and to briefly summarize main themes that appeared during the interviews.

The average time of interviews was 30 min. Considering the relatively homogeneous group of participants, data saturation was reached quickly, within the first ten participants. All recordings were depersonalized and coded by Yuliia Sereda. We collected the minimal set of demographic data, such as age, sex, position (manager, doctor, nurse), and years of experience. Demographic data were stored separately from the transcripts, and it did not include identifiers linking demographics with transcripts. We did not collect the names of participants or their health facilities and locations. Participants were provided the option to review their transcripts and provide feedback. None of the participants requested their transcripts for review.

#### 2.5.4. Data Analysis

Interviews were transcribed semi-verbatim. During data cleaning, we removed names of people or places from the transcripts when mentioned. MS Word transcripts were imported in RQDA, a package for qualitative data analysis in R software. We used the coding framework defined in the guide. Themes were explored question by question accounting for the category of participant (manager, doctor, or nurse). We did not identify themes in advance. We described participants’ demographics as aggregated numbers to prevent their identification by the individual demographic profile. We selected quotes that were illustrative of the points emphasized in the analysis, that were reasonably succinct, and that were representative of the patterns in the collected data. If contradictory evidence was found, we included several quotes representing confronting attitudes. We used generic signatures for selected quotes, such as ‘health manager’, ‘doctor’, or ‘nurse’. Participants did not provide feedback on the findings.

## 3. Results

### 3.1. Quantitative Study

#### 3.1.1. Patient Characteristics

In total, 844 patients with COVID-19 underwent Xpert MTB/RIF (Cepheid Inc., Sunnyvale, CA, USA) testing during the study period (Table 2). Most patients were urban residents (81.8%). Mean age was 43.5 ± 16 years, and about two-thirds of patients (64%) were male. Six patients (0.6%) were co-infected with HIV. Seven patients (0.8%) reported a history of TB treatment, and none of them were people living with HIV. None of the patients could identify their TB exposure at the time of COVID-19 diagnosis.

#### 3.1.2. TB Testing

On average, patients with COVID-19 received TB testing three days after the hospital admission (Table 3). Only one patient was not able to provide a sputum sample; however, bronchoalveolar lavage was collected.

Of 844 patients tested for TB, 47 patients (5.6%, 95% confidence interval (CI): 4.1–7.3%) received a positive result (MTB detected), including 13 patients with rifampicin resistance (1.5%, 95% CI: 0.8–2.6%). Ten out of forty-seven patients with MTB detected had a ‘trace’ result. Test results were invalid for two patients, and there was no follow-up testing. Culture-confirmed tuberculosis was found in 38 out of 47 (80.9%) patients with the positive Xpert MTB/RIF (Cepheid Inc., Sunnyvale, CA, USA). Culture was negative in 4 out of 13 patients with rifampicin resistance, 3 out of 24 patients with rifampicin sensitivity, and 2 out of 10 patients with a trace result. Patients with a ‘trace’ result (n = 10) were further evaluated and active TB was bacteriologically confirmed for all of them. Five out of ten patients with a ‘trace’ result were further diagnosed with rifampicin-resistant TB. As a result, the proportion of COVID-19 and rifampicin-resistant TB co-infection increased to 2.1%, 95% CI: 1.3–3.4% (18 patients). Overall, active TB diagnosis was laboratory (n = 45) or clinically (n = 2) confirmed in all patients who tested positive by Xpert MTB/RIF (Cepheid Inc., Sunnyvale, CA, USA).

All patients who reported previous history of TB (n = 7) tested positive by Xpert MTB/RIF (Cepheid Inc., Sunnyvale, CA, USA) (Table 4). Controlling for demographics and comorbidities, the proportion of COVID-19 and TB co-infection was higher in the Vicebsk region (adjusted proportion difference (aPD): 0.26, 95% CI: 0.19; 0.34, *p* < 0.001), among unemployed (aPD: 0.04, 95% CI: 0.001–0.08, *p* = 0.038), and among patients with co-morbidities (aPD: 0.04, 95% CI: 0.0004–0.08, *p* = 0.048).

#### 3.1.3. In-Hospital Mortality

Of 844 patients with COVID-19, 19 (2.3%) died during admission. The proportion of deaths was comparable in patients with and without confirmed TB co-infection (1/47, 2.1% (95% CI: 0.05–11.3%) and 18/797, 2.3% (95% CI: 1.3–3.6%), respectively, *p* > 0.99).

### 3.2. Qualitative Study

#### 3.2.1. Participant Characteristics

About half of the interview participants (6/13) were female. Age ranged from 27 to 59 years with a mean of 42 ± 11 years. On average, participants had 17 ± 12 years of experience in their position (min–max: 1.5–40).

#### 3.2.2. Relevance of TB Testing

All interviewed health workers considered laboratory TB testing among patients with COVID-19 as relevant. Compared to the nurses, managers and physicians were more aware of the reduction in notified TB cases during the COVID-19 pandemic, and believed that TB screening in patients with COVID-19 might help improve TB case-finding. They stressed that primary care facilities and general hospitals were overwhelmed with COVID-19 cases and tended to have low clinical suspicion of TB when patients present with clinical manifestations shared by two infections.

There was a general agreement that TB testing among patients with COVID-19 should be targeted. Participants named patients with severe COVID-19 and changes in lungs as priority groups for TB testing. Health workers cited the recent Order of the Ministry of Health in Belarus (October 2021) that recommends testing by Xpert MTB/RIF (Cepheid Inc., Sunnyvale, CA, USA) for all patients with COVID-19 who are admitted to repurposed departments of TB hospitals and have degenerative lung changes on radiography. Some TB physicians emphasized that laboratory TB testing is more relevant among people with TB-specific lung damages on images, notably middle and lower lung zones. There were opponents of this approach suggesting that differentiation between TB and COVID-19 related lung damage is not always possible. About half of the interviewed health workers supported the use of Xpert MTB/RIF (Cepheid Inc., Sunnyvale, CA, USA) among patients with COVID-19 diagnosis regardless of any lung changes, notably those with TB history or TB-specific symptoms at the time of COVID-19 diagnosis (unintentional weight loss, night sweats, coughing up blood, etc.) and representatives of TB key populations (people living with HIV, people who are immunocompromised, older people, prisoners, migrants, homeless people, etc.).

“*As a practitioner, I would support a more differentiated approach. We do need to conduct TB screening among patients with COVID-19, that’s without doubts. I just want to have a clear official document regulating who should be tested. GeneXpert is not cheap. There are financial constraints*”.

(TB hospital manager)

#### 3.2.3. TB Testing Procedures

Patients were informed about TB testing at the time of admission to the repurposed department of TB hospital by their physicians. Testing was considered as part of a general clinical examination, and patients were not requested to provide a separate informed consent for the procedure. On the day of the testing, nurses who were responsible for sputum collection described the testing procedures to the patients. According to the interviewed health workers, patients rarely refused to get tested. Testing fears and worries were related to TB stigma, notably a belief that TB affects only socially deprived populations. In this case, additional counseling was provided by a TB physician or a psychologist.

“*Some patients are indifferent. Well, it [Xpert MTB/RIF] is just another test. “Doctor, do what you have to do, I feel so bad”. Some people are concerned. They consider COVID-19 as a relatively “good disease” while TB is a “bad disease”. They say: “Why? I have never had TB. I’m not homeless. I’m not an alcoholic. Why should I do it?”. In this case, we explain that Belarus is endemic for TB*”.

(TB hospital manager)

Participants described sputum collection procedures in line with the national standards. All facilities had special rooms and trained staff to assist with sputum collection. However, sputum collection rooms were not always a part of the ‘red zone’, a pathway designed for patients with COVID-19. As a result, sputum could be collected in a treatment unit shared by several patients. Sputum collection in the treatment unit was common among patients with severe COVID-19, who were oxygen-dependent and had low mobility capacity.

“*In TB hospitals, it is a well-established algorithm. We have a sputum collection room with an engineering level of infection control, such as mechanical ventilation, and personal protection equipment for staff. We use respirators and protective suits. The laboratory is in the same building. Usually, we receive results within a day*”.

(TB hospital manager)

“*If the patient is oxygen dependent, and 99% of our patients [with COVID-19] are oxygen dependent…if we transport them to the sputum collection room, which is located in another building block, and back, we could make them worse*”.

(TB Doctor)

Sputum induction by inhalation of an aerosol of hypertonic saline helps patients cough up secretions from the lungs. Considering the high risk of airborne transmission of respiratory pathogens, an isolation room is recommended for the procedure. During interviews, health workers shared that sputum induction could be conducted in the treatment unit. In some repurposed departments, particularly intensive care units, up to 50% of the patients with severe COVID-19 could not produce sputum without induction. Transportation of these patients to an airborne infection isolation room was not always possible.

All TB hospitals were equipped with laboratories and physicians received test results within a day. Participants described logistics of sputum transportation to the laboratory according to local safety protocols. Health workers participating in the interviews did not report any side effects during TB testing among patients with COVID-19.

#### 3.2.4. Patient’s Pathway after the Bacteriological TB Confirmation

Most patients with laboratory-confirmed COVID-19 and TB co-infection were transferred to the TB department within the same hospital—a special unit for patients with COVID-19 and TB co-infection or individual rooms. Respondents pointed out that it was easier to find an individual room in the TB department rather than in the repurposed department for COVID-19, considering the reduction in TB notifications. In some hospitals, patients remained in the treatment unit the end of their COVID-19 treatment if it was an individual room. Practices of co-infection treatment management varied between different regional hospitals. In hospitals, where TB physicians and nurses worked in both COVID-19 and TB departments, patients could continue to be managed by the physician appointed for COVID-19 treatment even after their transfer to the TB department. If there was no overlap in the staff across departments, TB physicians from the TB department managed the co-infection treatment on their own or with a guidance of a Pulmonologist/Infectious Diseases Physician from the COVID-19 department.

“*According to the infection control measures, we transfer a patient with the diagnosis [of COVID-19 and TB co-infection] to the department for drug-sensitive or drug-resistant TB. We have these separated. Patients remain in an isolation ward, with their own bathroom. In 2020, all TB physicians in our hospital were remotely trained on coronavirus infection. Therefore, they can treat the co-infection together with Pulmonologists, and on their own.*”

(TB Nurse)

#### 3.2.5. Barriers of TB Screening

Study participants believed that TB testing at TB hospitals is a ‘debugged system’, and repurposed departments do not experience any barriers related to staff, equipment, or logistics. Health workers who worked at the facilities where sputum collection rooms were not a part of ‘a red zone’ reported this as a single barrier. When asked about testing worries and fears among patients, physicians and nurses believed that it is not a barrier. From their experience, additional counseling fully resolves the issue, and all patients with testing anxiety were tested after the additional counseling was done. Staff at repurposed departments of TB hospitals were motivated to continue laboratory TB testing among patients with COVID-19. Representatives of the National TB Programme stressed that implementation of the intervention was feasible because it did not require additional human or financial resources.

“*We expected to diagnose and treat more patients than we have now. TB resources are underused. Xpert MTB/RIF cartridges and anti-TB drugs have limited shelf-life. We can waste resources if a decrease in TB detection will continue. TB hospitals do not need additional training to implement Xpert MTB/RIF testing among patients with COVID-19. There is no need for additional funding. With this intervention, we will use resources more efficiently.*”

(Representative of the National TB Programme)

Integration of TB screening in repurposed departments of other facilities might be challenging. Respondents suggested the scale up of Xpert MTB/RIF (Cepheid Inc., Sunnyvale, CA, USA) among patients with COVID-19 admitted to Infectious Diseases Hospitals at facilities where staff have been trained in sputum collection. When asked about obstacles for the TB screening in patients with COVID-19 in other health facilities, respondents named the limited number of nurses trained in sputum collection, lack of motivation among health workers, shortage of GeneXpert Systems and longer turnaround time to obtain test results. Physicians emphasized that it is critical to have an Order of the Ministry of Health recommending laboratory TB testing among hospitalized patients with COVID-19 in other facilities, similar to the one developed for repurposed departments of TB hospitals.

“*Of course, we have to scale up [Xpert MTB/RIF testing among patients with COVID-19] in other hospitals. However, there is one problem, they don’t have enough trained staff. Sputum collection is not a routine procedure there. Secondly, they often don’t have enough sputum collection rooms. Plus, logistics. Not all hospitals have GeneXpert. Transportation of a dangerous biological sample, considering COVID-19 and TB, is another issue.*”

(Representative of the National TB Programme)

## 4. Discussion

The COVID-19 pandemic decreased TB detection through a shift in the healthcare priorities and diversion of resources. Our study addressed the question of whether TB screening among patients with COVID-19 is feasible and can help identify missed TB cases. The Belarus National TB Programme, together with the WHO Regional Office for Europe, explored outcomes of Xpert MTB/RIF (Cepheid Inc., Sunnyvale, CA, USA) testing among patients admitted to repurposed departments of TB hospitals for COVID-19 treatment.

In our study, one in twenty patients (5.6%) with COVID-19 requiring hospitalization were found to be co-infected with active pulmonary TB. This result was higher than the findings of a recent meta-analysis from 43 countries (mostly from Africa and Asia), where pooled prevalence of active pulmonary tuberculosis was about 1.1% among all patients with COVID-19 and 3.9% among patients with severe COVID-19 who received inpatient treatment [14].

About one in five patients with COVID-19, who tested positive for TB, had a history of prior TB treatment or were co-infected with HIV. HIV infection is a well-known risk factor for TB due to immunosuppression [15]. Multiple comorbidities, such as HIV and COVID-19, often lead to more compromised immunity, which increases the susceptibility for TB. People with COVID-19 and recurrent TB could have endogenous reactivation or exogenous reinfection. Immunosuppression is independently associated with both types of recurrent TB [16]. Genetic, microbiological, and social factors also contribute to TB reinfection. Immune mechanisms necessary for suppression of TB may not be fully restored after successful TB treatment [17]. TB reinfection is more likely among patients with the Beijing genotype of TB strains and a history of immigration and imprisonment [16]. However, our study did not collect information on these characteristics.

As compared to other study sites, Vicebsk’s TB hospital had a considerably higher proportion of patients with COVID-19 and TB co-infection, controlling for other patient characteristics. About one-third of admitted patients with COVID-19 tested positive for active TB there. This finding is unexpected considering that Vicebsk is not a region with high TB burden compared to other study sites. The National TB Programme conducted an investigation in Vicebsk and came to a conclusion that the region experienced a TB outbreak at the time of data collection, which was related to partial interruption of TB screening services in a few months preceding the intervention. Contrary to the national trend, TB notifications started to increase in the region since August 2021, a month before the intervention was implemented. There was no evidence of preferential testing, i.e., only among patients with a high level of TB suspicion.

We did not find a higher risk of in-hospital mortality in patients with TB and COVID-19 co-infection as compared to patients without TB co-infection. It is inconsistent with the findings of systematic reviews, which showed that patients with concurrent TB and SARS-CoV-2 infection are prone to poor outcomes [14,18]. Overall, in-hospital mortality was low in our cohort (2%). Rates of COVID-19 in-hospital mortality vary dramatically across these studies, which is partially explained by characteristics of cohorts, notably severity of disease and prevalence of comorbidities [19].

The qualitative component of our study examined attitudes on laboratory TB screening among patients with COVID-19 among health workers involved in the intervention. A general theme emerged that TB screening should be differential, targeting certain groups of patients with COVID-19. While recommendations of respondents were in line with the WHO guideline on TB screening among people with shared symptoms, exposures and risk factors for two diseases, there was a demand for more specific criteria considering constrained resources. The quantitative study suggests that COVID-19 and TB co-infection was more common among unemployed and patients with comorbidities. Belarus may prioritize these groups for joint COVID-19 and TB screening. In this regard, bi-directional algorithm of COVID-19 and TB screening in India is of interest [20]. India screens for TB all patients with confirmed COVID-19 using a four symptoms questionnaire (cough > 2 weeks, fever > 2 weeks, weight loss, night sweats). Patients experiencing any of these symptoms are examined by chest X-rays and those with any lung abnormalities receive a TB test.

Individual interviews highlighted several challenges of infection control during TB testing among patients with COVID-19, such as a lack of sputum collection room in-hospital zones repurposed for treatment of COVID-19 or inability to transport patients with severe illness to these rooms. While aerosol-generating procedures are generally not recommended due to their potential infection risk to healthcare providers and other people [21], our findings showed that sputum induction was common in patients with severe COVID-19.

Our study was restricted to TB hospitals, which are better equipped for TB testing compared to other health facilities. Several barriers of scaling up Xpert MTB/RIF (Cepheid Inc., Sunnyvale, CA, USA) among patients receiving COVID-19 treatment in other hospitals were mentioned in the interviews, such as limited human and financial resources, lack of legislation regulating service implementation, and training needs. Implementation barriers were consistent with those reported in the literature [22].

There are several important limitations to the study. The study population represented patients admitted for inpatient treatment for COVID-19 in TB hospitals, which may limit the generalizability and transportability of findings across Belarus and other countries. In Belarus, patients with COVID-19 requiring hospitalization were admitted to TB hospitals based on proximity criteria (the nearest health facility with inpatient COVID-19 treatment) or when other hospitals experienced shortage of beds. The study did not include asymptomatic COVID-19 patients and symptomatic COVID-19 patients receiving outpatient treatment. Therefore, we might have overestimated the true prevalence of COVID-19 and TB co-infection. Our study was restricted to pulmonary TB diagnosis, and we did not assess the co-infection of COVID-19 and extrapulmonary TB. A general weakness of the in-depth interviews is that the quality of results depends highly on the interviewee’s willingness to provide truthful information. Sampling bias could occur for the selection of the participants for the individual interviews. The limitations of remote interviews include digital literacy, reduction of social cues, and challenges in maintaining connection, amid a lack of control over the participant’s physical environment, its security, and privacy.

This is the first study assessing the proportion of pulmonary TB among patients with COVID-19 in Europe to the best of our knowledge. Interventions on TB screening among patients with COVID-19 remain rare in TB-endemic countries. The strengths of our study include a relatively large cohort of patients and a combination of quantitative and qualitative methods. While the study objectives did not include the follow-up of patients after receiving Xpert MTB/RIF result, we conducted a sensitivity analysis to retrospectively examine whether positive results were further confirmed by sputum culture or other examinations.

Our study has several implications. TB screening of patients with COVID-19 at repurposed departments of TB hospitals identified many new TB cases. Most patients who tested positive for TB did not have a history of prior TB and were not co-infected with HIV. Thus, they were not subjected to the routine periodic TB screening implemented in Belarus. It is likely that their TB disease would be diagnosed at later stages. The early detection of TB and COVID-19 co-infection is very important for the prevention of contact transmission, and proper management of both diseases. As an implication of the operational research, a Ministry of Health Order was approved that regulates TB testing among patients with COVID-19 admitted to repurposed departments of TB hospitals. When the National TB Programme initiated TB testing among patients with COVID-19, the intervention was recommended to TB hospitals and implementation was optional. With a new order, all TB hospitals accepting patients with COVID-19 were engaged in the intervention. This study identified gaps and barriers in TB testing among patients with COVID-19 that will inform further implementation of the intervention and a potential scale-up in other hospitals. More efficient use of TB resources is another implication. With a reduction in TB notifications, Xpert MTB/RIF (Cepheid Inc., Sunnyvale, CA, USA) cartridges are underused. Furthermore, fewer TB diagnoses led to a decrease in uptake of anti-TB drugs. TB screening among patients with COVID-19 at TB hospitals did not impose additional financial burden and allowed to utilize resources that could be unexploited otherwise. Considering implications for future research, the promise of laboratory TB testing among patients with COVID-19 should be evaluated, such as the number needed to screen and cost-effectiveness of the screening algorithm.

## 5. Conclusions

Nearly one in twenty patients with COVID-19 who had lung changes and required hospitalization in Belarus were co-infected with active pulmonary TB, and over one-third of them had rifampicin resistance. The qualitative study has demonstrated that it is feasible to deliver laboratory TB testing among patients with COVID-19 at repurposed departments of TB hospitals considering available human, material, and financial resources. The intervention may help to improve TB case-finding during the COVID-19 pandemic and can be recommended for the routine practice in Belarus and similar settings with high TB burden.

## Figures and Tables

**Table 1:** Characteristics of the study sites.

	Homel’ Region	Hrodna Region	Mahilëu Region	Minsk City	Vicebsk Region
Notified TB cases, n (rate per 100,000 population)					
2019	373 (27)	208 (20)	248 (24)	180 (9)	171 (15)
2020	260 (19)	127 (12)	167 (16)	141 (7)	139 (12)
Extrapulmonary TB cases, n (%)					
2019	54 (14)	12 (6)	2 (1)	21 (12)	4 (2)
2020	25 (10)	5 (4)	7 (4)	13 (9)	4 (3)
Total beds in TB hospitals, n	310	301	295	300	210
Beds in TB hospitals repurposed for patients with COVID-19, n (%)	60 (19)	126 (42)	60 (20)	100 (33)	120 (57)

**Table 2:** Characteristics of patients with COVID-19 admitted to repurposed departments in TB hospitals, Belarus, April–November 2021.

Characteristic	N	%
Total	844	100
Site		
Minsk city	241	(28.6)
Hrodna	484	(57.3)
Homel’	65	(7.7)
Vicebsk	43	(5.1)
Mahilëu	11	(1.3)
Age in years, mean (standard deviation)	43.5	(15.6)
Sex, n (%)		
Male	540	(64.0)
Female	304	(36.0)
Residence, n (%)		
Urban	690	(81.8)
Rural	154	(18.2)
Employment, n (%)		
Employed	455	(53.9)
Unemployed	222	(26.3)
Other (disable, maternity leave, housekeeper, student)	167	(19.8)
Had history of TB treatment, n (%)	7	(0.8)
Had contact with TB prior to admission, n (%)	0	(0)
BMI, n (%)		
Underweight	97	(11.5)
Healthy weight	522	(61.8)
Overweight	164	(19.4)
Obesity	61	(7.2)
Comorbidities, n (%) ^a^		
HIV	6	(0.7)
Diabetes	48	(5.7)
Other:	270	(32)
Circulatory system diseases	109	(12.9)
Ischemic heart diseases	81	(9.6)
Hypertensive diseases	75	(8.9)
Digestive system diseases	26	(3.1)
Endocrine, nutritional or metabolic diseases	21	(2.5)
Lower respiratory tract diseases	19	(2.3)
Chronic kidney disease	14	(1.7)
Cerebrovascular disease	12	(1.4)
Liver diseases	10	(1.2)
Oncological diseases	9	(1.1)
Length of stay in TB hospital in days, median [interquartile range]	16	[14–19]
Days between COVID-19 diagnosis and admission to TB hospital, median [interquartile range]	1	[0–3]
Outcome at discharge, n (%)		
Cured/Improved	779	(92.3)
Transferred	46	(5.5)
Died	19	(2.3)

^a^ Patients could have several comorbidities.

**Table 3:** Characteristics of laboratory TB testing among patients with COVID-19 admitted to repurposed departments in TB hospitals, Belarus, April–November 2021.

Characteristic	n	%
Total	844	100
Days between admission to TB hospital and Xpert MTB/RIF testing, median [interquartile range]	3	[2–5]
Type of sample for Xpert MTB/RIF testing		
Sputum	843	(99.9)
Bronchoalveolar lavage	1	(0.1)
Result of Xpert MTB/RIF testing		
Negative	795	(94.2)
Positive	47	(5.6)
Rifampicin-resistant	13	(1.5)
Rifampicin-sensitive	24	(2.8)
Trace	10	(1.2)
Invalid	2	(0.2)

**Table 4:** Factors associated with laboratory-confirmed COVID-19 and TB co-infection among patients admitted to repurposed departments in TB hospitals, Belarus, April–November 2021 (among patients with valid test results, N = 842).

Characteristic	MTB Detected (N = 47) n (row %)	MTB Not Detected (N = 795) n (row %)	Unadjusted Proportion Difference, 95% CI, *p*-Value	Adjusted Proportion Difference, 95% CI, *p*-Value
Site				
Minsk city	7 (2.9)	233 (97.1)	ref.	ref.
Hrodna	23 (4.8)	461 (95.2)	0.02 [−0.02; 0.05] *p* = 0.297	0.01 [−0.03; 0.05] *p* = 0.614
Homel’	4 (6.2)	60 (93.8)	0.03 [−0.03; 0.09] *p* = 0.288	0.03 [−0.03; 0.10] *p* = 0.315
Vicebsk	**13 (30.2)**	**30 (69.8)**	**0.27 [0.20; 0.35] *p* < 0.001**	**0.26 [0.19; 0.34] *p* < 0.001**
Mahilëu	0 (0)	11 (100)	NA	NA
Age in years				
18–34	9 (3.2)	273 (96.8)	ref.	ref.
35–44	12 (6.8)	164 (93.2)	0.04 [−0.01; 0.08] *p* = 0.100	0.03 [−0.01; 0.07] *p* = 0.160
45–54	**15 (8.5)**	**161 (91.5)**	**0.05 [0.01; 0.10] *p* = 0.016**	0.04 [−0.01; 0.08] *p* = 0.119
>54	11 (5.3)	197 (94.7)	0.02 [−0.02; 0.06] *p* = 0.317	−0.02 [−0.07; 0.03] *p* = 0.495
Sex				
Male	36 (6.7)	503 (93.3)	0.03 [0.00; 0.06] *p* = 0.065	0.03 [−0.01; 0.06] *p* = 0.119
Female	11 (3.6)	292 (96.4)	ref.	ref.
Residence				
Urban	34 (4.9)	654 (95.1)	ref.	ref.
Rural	13 (8.4)	141 (91.6)	0.03 [−0.01; 0.08] *p* = 0.087	0.02 [−0.02; 0.07] *p* = 0.261
Employment				
Employed	19 (4.2)	435 (95.8)	ref.	ref.
Unemployed	20 (9.0)	201 (91.0)	**0.05 [0.01; 0.09] *p* = 0.01**	**0.04 [0.001; 0.08] *p* = 0.038**
Other (disable, maternity leave, housekeeper, student)	8 (4.8)	159 (95.2)	0.01 [−0.03; 0.05] *p* = 0.77	0.01 [−0.04; 0.06] *p* = 0.790
BMI				
Underweight	7 (7.2)	90 (92.8)	0.02 [−0.03; 0.07] *p* = 0.424	0.03 [−0.02; 0.08] *p* = 0.270
Healthy Weight	27 (5.2)	494 (94.8)	ref.	ref.
Overweight/Obese	13.0 (5.8)	211 (94.2)	0.01 [−0.03; 0.04] *p* = 0.735	−0.01 [−0.04; 0.03] *p* = 0.718
Had history of TB treatment ^a^				
Yes	7 (100)	0 (0)	Not applicable	-
No	40 (4.8)	795 (95.2)	ref.	-
HIV ^a^				
Yes	2 (33.3)	4 (66.7)	**0.28 [0.10; 0.46] *p* = 0.003**	-
No/Not tested	45 (5.4)	791 (94.6)	ref.	-
Diabetes ^a^				
Yes	2 (4.2)	46 (95.8)	−0.02 [−0.08; 0.05] *p* = 0.661	-
No	45 (5.7)	749 (94.3)	ref.	-
Any comorbidities				
Yes	23 (7.9)	269 (92.1)	**0.04 [0.003; 0.07] *p* = 0.035**	**0.04 [0.0004; 0.08] *p* = 0.048**
No	24 (4.4)	526 (95.6)	ref.	ref.

^a^ Variables were not included in the adjusted analysis considering the low number of patients in some categories. Bold font represents statistically significant findings (*p*-value ≤ 0.05).

## Data Availability

The data that support the findings of this study are available from the corresponding author, Oleksandr KOROTYCH, upon reasonable request.

## Supplementary Materials

The following supporting information can be downloaded at: https://www.mdpi.com/1660-4601/19/7/4370/s1, Table S1: Guide for individual interviews with health care workers involved in Xpert MTB/RIF testing among patients with COVID-19 requiring hospitalization in Belarus.
