# Supplementary material for: Tuberculosis Co-Infection Is Common in Patients Requiring Hospitalization for COVID-19 in Belarus: Mixed-Methods Study
Source: Int J Environ Res Public Health. 2022 Apr 5;19(7):4370. doi: 10.3390/ijerph19074370 (PMC9028713; doi:10.3390/ijerph19074370)
Supplement: Supplementary file 1 [file ijerph-19-04370-s001.zip › ijerph-1619930-supplementary.pdf]

Supplementary Material

Table S1: Guide for individual interviews with health care workers involved in Xpert MTB/RIF testing among patients with COVID-19 requiring hospitalization in Belarus

Question	Probes	Thematic code
1. How COVID-19 outbreak and related interventions have influenced TB screening in Belarus?	None	relevance – general
2. How relevant is TB screening to the people diagnosed with COVID-19?	Relevant / not relevant<br>What groups of people diagnosed with COVID-19 should be tested (i.e., all, severe cases, elderly etc.)?	relevance - population
3. What are the documents outlining TB screening in patients with COVID-19 in. Belarus?	National protocols / guidelines<br>Facility-level documents	guidelines
4. How patients with COVID-19 were informed about Xpert MTB/RIF testing in your facility?	For whom the test was offered<br>Who managed initial counselling?<br>What information was shared with patients?	offer
5. Could you please describe in detail how Xpert MTB/RIF testing was conducted in patients with COVID-19 in your facility?	Testing algorithm<br>Sample collection setting<br>Staff involved<br>Sample delivery to the laboratory<br>Result notification	testing logistics
6. What is the further pathway for the patient to confirm TB diagnosis and initiate treatment in your facility?	Diagnosis algorithm<br>Staff involved (Can health workers managing COVID-19 treatment in TB hospitals confirm TB diagnosis?)<br>Do patients remain in the same room after confirming COVID-19/TB co-infection?	diagnosis
7. Can you please tell me about your thoughts and experience how patients with COVID-19 reacted to Xpert MTB/RIF testing in your facility?	Interest in testing<br>Questions asked by patients about testing<br>Refusals, possible explanations/causes for patients refusing testing<br>Possible facilitators for patients to do testing	perceived patient views
8. What is the level of safety of the procedures in the Xpert MTB/RIF testing among patients with COVID-19?	Infection control<br>Any unexpected adverse events	safety
9. Based on your experience, what could be done differently to manage Xpert MTB/RIF testing in TB hospitals?	Infrastructure<br>Staff<br>Sample collection<br>Sample delivery to the laboratory<br>Result notification	service delivery recommen-dations
10. In your opinion what are the main challenges/obstacles for the TB screening in patients with COVID-19 in TB hospitals?	Absence of clear policy and guidelines<br>Lack of knowledge of specialists	barriers – TB facilities

	Lack of health personnel or unmotivated personnel<br>Lack/or problems with infrastructure<br>Lack of funding	
11. What do you think about integrating TB screening to other health facilities providing diagnosis and treatment services for patients with COVID-19?	What facilities should provide TB screening in addition to COVID-19 diagnosis and treatment<br>How TB screening should be managed there	other facilities – feasibility
12. In your opinion what would be the main challenges/obstacles for the TB screening in patients with COVID-19 in other health facilities?	See #10<br>Discuss the following health facilities: primary health care / polyclinics and infectious disease hospitals.	other facilities – barriers
13. What do you think about sustainability of TB screening in patients with COVID-19 in TB hospitals?	Willingness of staff to continue TB screening<br>Available resources<br>Whether TB hospitals will continue to admit patients with COVID-19	sustainability and intent to continue use
14. Any other important issues related to TB screening in patients with COVID-19 you would like to mention?	None	closing remarks
